# Assessing Patient Organization Participation in Health Policy: A Comparative Study in France and Italy

**DOI:** 10.15171/ijhpm.2017.44

**Published:** 2017-04-15

**Authors:** Kyriakos Souliotis, Eirini Agapidaki, Lily Evangelia Peppou, Chara Tzavara, Dimitrios Varvaras, Oreste Claudio Buonomo, Dominique Debiais, Stanimir Hasurdjiev, Francois Sarkozy

**Affiliations:** ^1^ Faculty of Social and Political Sciences, University of Peloponnese, Corinth, Greece.; ^2^ Community Mental Health Centre, University Mental Health Research Institute, Athens, Greece.; ^3^ Department of Surgery, University of Rome Tor Vergata, Rome, Italy.; ^4^ Europa Donna France, Paris, France.; ^5^ Bulgarian National Patients’ Organization, Sofia, Bulgaria.; ^6^ FSNB Health & Care, Paris, France.

**Keywords:** Patient Participation, Health Policy Decision-Making, Cancer Patient Organizations (CPOs), Scale Development

## Abstract

**Background:** Even though there are many patient organizations across Europe, their role in impacting health policy decisions and reforms has not been well documented. In line with this, the present study endeavours to fill this gap in the international literature. To this end, it aims to validate further a previously developed instrument (the Health Democracy Index - HDI) measuring patient organization participation in health policy decision-making. In addition, by utilizing this tool, it aims to provide a snapshot of the degree and impact of cancer patient organization (CPO) participation in Italy and France.

**Methods:** A convenient sample of 188 members of CPOs participated in the study (95 respondents from 10 CPOs in Italy and 93 from 12 CPOs in France). Participants completed online a self-reported questionnaire, encompassing the 9-item index and questions enquiring about the type and impact of participation in various facets of health policy decisionmaking. The psychometric properties of the scale were explored by performing factor analysis (construct validity) and by computing Cronbach α (internal consistency).

**Results:** Findings indicate that the index has good internal consistency and the construct it taps is unidimensional. The degree and impact of CPO participation in health policy decision-making were found to be low in both countries; however in Italy they were comparatively lower than in France.

**Conclusion:** In conclusion, the HDI can be effectively used in international policy and research contexts. CPOs participation is low in Italy and France and concerted efforts should be made on upgrading their role in health policy decision-making.

## Introduction

Patient participation is a multidimensional concept originating from the widespread consumer movement in 1960’s, which was centered on consumers’ right to safety, to be informed, to choose and to be heard.^1^ Nonetheless, the concept is considered ill-defined due to diversity of its applicability^2^ and various terms, such as patient collaboration, patient involvement, partnership, patient empowerment or patient-centered care, have been used interchangeably in existing literature.^2^ Moreover, patient participation pertains to a wide range of healthcare aspects, such as decision-making, self-medication, self-monitoring, patient education and goal setting, among others. In this rationale, patient participation is conceptualised as the process that allows patients to be an integral part of the decision-making course that influences their health.^3,4^ Congruent with this, they can contribute to a wide range of activities from formulating treatment plans to shaping germane policies.

Patient participation in developing health policies refers to individuals’ involvement in every step of planning, development and implementation of healthcare programs, interventions and services.^5,6^ This approach implies that patient participation may occur on various levels: (*i*) the individual or micro level, wherein the patient participates as a member of the healthcare team in decisions that directly affect his/her health (eg, clinical decision-making); (*ii*) the meso level, which refers to engagement in health decision-making on a local or organizational level (eg, local health authorities and/or hospital organizations), and (*iii*) the macro level, which involves participation in health issues on a federal, national or international level.^7-10^ The importance of patient involvement in every stage of the healthcare process has been substantiated.^11,12^ There are 2 lines of argumentation justifying patient organization participation in health policy decision-making. The first is political and stems from the democratic values of transparency and accountability^13^; the second is health-related and derives from the principle of equity and the health promotion approach.^14^ Patients are supported to increase the control over the factors which influence their health and become more active in the development, planning, delivery and evaluation of health services.^15^ Indeed, existing research indicates that increased patient participation is linked to enhanced quality of healthcare services, elevated effectiveness and efficiency of health systems and better population health outcomes.^16-19^ On the other hand the sustainability and effectiveness of the healthcare systems is threatened due to the high prevalence of the non-communicable diseases (NCDs) worldwide. The high prevalence of NCDs has resulted in growing demands and costs due to the manifold impact of chronic illnesses on health and healthcare.^20,21^ Patient participation may contribute to a more effective allocation of resources so as to ensure that effective and sustainable health services will be provided.

As a corollary of this, a lot of programs have been implemented internationally, geared towards raising patients’ awareness and empowerment. In this way, patients may participate more actively and effectively in health decision-making processes. These initiatives have largely focused on improving health literacy, enhancing patient involvement in treatment decision-making and fostering self-management support. Consistent with this, treatment options become individualized and tailored to a patient’s profile, strengthening therefore his or her motivation.^10,22-25^ Since the primary goal of these programs is to achieve changes on the micro-level, the main research activity is concentrated on the micro-level. On the other hand, initiatives at meso and macro levels are scarce and essentially serve advocacy purposes.^26-28^

Similarly, although there are instruments, albeit poorly validated,^29^ assessing patient participation on the micro-level, there is a paucity of tools for gauging the degree of patient participation on the meso and macro level.^10,17,29^

Patients suffering from chronic diseases usually seek additional information and social support in several formal and informal patient organization groups. There are different types of patient groups and networks, targeting a diversity of patient needs.^30^ For instance, many cancer patients seek social support in groups created so as to strengthen the social network of cancer patients and to empower their caregivers^31^; while others prefer to join a formal cancer patient organization (CPO) to acquire more information about the healthcare system and the reimbursement processes. In the second case, patients become familiar with health policy strategies and processes in the majority of instances.^32^ Thereby, patients advance their understanding about health policy decision- making and thus claim equal participation.^33^ Even though there are many patient organizations across Europe, their role in impacting health policy decisions and reforms has not been well documented.^34^

The limited number of qualitative studies in the field emanate predominantly from the United Kingdom, the United States, Germany and the Netherlands.^30,35-38^ Converging evidence from these countries substantiates a proliferation of patient organizations during the past decades and increased contacts between patient groups and policy-makers.^30,35-38^ In fact, Allsop and colleagues report that professionals bodies and pharmaceutical companies have increasingly included patient groups in discussions on policy proposals in the United Kingdom.^38^ Nonetheless, the growing participation of patient groups in health policy does not necessarily lead to their political effectiveness, with some authors arguing that it constitutes mere incorporation rather than effective participation.^38^ Similarly, research in the Netherlands indicates that while the system provides many opportunities for patient organizations to participate, patient groups often lack the resources or/and the expertise to meet pertinent demands.^30^ As a corollary of this, they cannot be an equal party in health policy process. This strand of research provides very valuable insights into patient groups and their political effectiveness in these countries; however, these studies have primarily employed qualitative methodology to meet their research aims. Up to date, no quantitative study has taken place to explore patient organization participation in health policy in 2 European countries. The aim of the present study is to fill this gap in the international literature, by exploring the influence of CPOs in health policy-decision-making in 2 European countries.

The reason for concentrating on cancer rather than on other chronic or debilitating health conditions pertains to evidence suggesting that cancer remains a key public health concern, incurring substantial burden to European societies.^39^ In particular, it constitutes one of the main causes of morbidity and mortality worldwide, while the cost of treatment remains high.^40^ There is also a large body of literature corroborating an association between inequalities in accessing cancer treatment and elevated risk of mortality^41,42^; while overcoming barriers to cancer treatment is top priority.^41^ In line with these, CPOs may play a prominent role in facilitating removal of these barriers, advocating patients’ rights and influencing germane policies.

Consistent with the aforementioned, the present study has set out the following objectives:

(*i*) to validate further a previously developed instrument (the Health Democracy Index - HDI) for measuring CPO’s degree and impact of participation in health policy decision-making by utilizing this tool;

(*ii*) to compare the degree and impact of CPOs in health policy in Italy and France.

A previous exploration of the HDI in a random sample of members of patient organizations in Greece indicated that the scale has good psychometric properties: construct and convergent validity, internal consistency and test-retest reliability.^43^ This is the first attempt to adapt the instrument to another language and culture.

## Methods

### Instrument Adaptation

The HDI is an original scale consisting of 8 items, enquiring about patient organization participation in health policy decision processes at the meso- and macro-level: reforms, panels at the Ministry of Health, panels in other prominent health-related organizations, hospital boards, Ethics Committees in clinical trials, health technology assessment procedures and the national parliament. For each item, 7 response options were provided: (*i*) it is not a legal requirement and it never happens, (*ii*) ) it is not a legal requirement and it rarely happens, (*iii*) it is not a legal requirement but it often happens, (*iv*) it is a legal requirement and it never happens, (*v*) it is a legal requirement and it often happens, (*vi*) it is a legal requirement and it happens very often, and (*vii*) it is a legal requirement and it always happens. In addition, there was one question enquiring about the frequency whereby a substantial change in a health policy decision was evoked as a result of the involvement of patient organization in the process. For this item, ratings were made on a 7-point scale reflecting a frequency dimension: never-very rarely-rarely-sometimes-often-very often. Higher composite scores indicated higher levels of patient organisation participation.

The development of the index^43^ followed the subsequent steps: (*i*) definition of the construct, (*ii*) review of the construct definition, (*iii*) item drafting, (*iv*) item review, and (*v*) pilot testing of its psychometric properties (reliability: internal consistency, test-retest reliability and validity: construct validity and convergent validity. Furthermore, weights were assigned to the items of the index by employing a Delphi methodology.

After the positive results of the pilot study,^43^ the research group started processing participants’ feedback on the instrument. Moreover, an international working group, consisting of European stakeholders (policy-makers, members of patient organizations and researchers with background in patient empowerment), held various meetings to discuss the adaptation of the index to European standards. As a corollary of these, it was decided to divide the item exploring health technology assessment procedures into 2 components: scientific assessment and economic assessment, with an accompanying division of their weights as well. In this reasoning, the HDI became a 9-item index. For each of the nine questions of the HDI, one item enquiring about the type of patient organization participation and one concerning its impact were added. The type of patient participation was rated as “observer” (mere presence of the member/non-voting), “consulting” (the member provides information and/or makes recommendations on a particular subject, non-voting), “vocal participant” (the member delivers a presentation and/or makes a speech, non-voting), “voting member.” The item capturing the impact of this participation was rated on a 6-point scale ranging from “absent” to “very high.”

Apart from the HDI, the research instrument encompassed the following sub-sections: respondents’ socio-demographic characteristics, respondents’ disease characteristics (eg, familiarity with disease, year of initial diagnosis, knowledge of treatment options), 4 items about the individual’s involvement in the patient organization (eg, position in the organization, years of membership, degree of personal involvement) and 9 items assessing the degree of participation and impact of all patient organizations in the country (eg, the degree of participation/impact/collaboration of all patient organizations in health policy decision-making in the country).

The instrument was translated and back-translated from Greek to English by 2 bilingual researchers. A similar process was then followed for translation and back translation from English to French and Italian.**

### Sample and Procedure

A convenient sample of 188 patients-members of CPOs were included in the study. Potential participants were identified through contact with the CPOs. A CPO was defined as any patient group with a legal entity, (official associations), that focused on cancer. To be eligible for inclusion into the study, CPOs had to: (*a*) be active on a national level, (*b*) represent cancer patients, and (*c*) have an accessible website.

A total of 43 CPOs, which fulfilled the inclusion criteria in France, and 27 in Italy were invited to participate in the study. Since an official list of European Union (EU) patient organizations does not exist and thus the number of CPOs in each country as well as the number of members of each CPO is inaccessible, we endeavoured to minimize selection bias by identifying eligible CPOs through different sources: online databases, registries of European websites relevant to cancer patient groups, lists belonging to the Ministry of Health, contacts with key persons in each country and European umbrella organizations. After contacts were made, 10 CPOs in Italy (37%) and 12 in France (28%) agreed to take part. In Italy, 8 CPOs (30%) did not reply to repeated calls and emails of the research team and 9 (33%) refused to participate. Similarly, in France, 15 CPOs did not respond to the calls/emails (37%) and 16 (37%) refused to participate. Reasons for refusal include: they considered participation time consuming, other activities were in priority at the time, the CPO was not particularly active during the past year and difficulties with the Review Board that had to approve the study protocol. Upon comparing CPOs that agreed to participate and those that did not, one may discern that the most visible and active CPOs participated.

For the recruitment of potential participants, an email invitation was sent to the president or other contact person of the CPO. After initial acceptance of participation, the Institutional Review Board of each organization approved the study protocol. Data was collected online. Board members of each patient agency circulated an invitation for participation to all members of the organization. If a member agreed to participate and signed the written informed consent, the board members would forward the questionnaire either via email or a web-link. In order to be eligible for the study, patients should have been adult members of the CPO, with sufficient knowledge of Italian/French or English language. A total of 95 members from the 10 CPOs in Italy and 93 from the 12 CPOs in France provided complete data. Data were collected the time period January to February 2016.

Sample characteristics can be found in Table 1. As shown in Table 1, no statistically significant differences emerged between respondents in Italy and France.

**Table 1:** Respondents’ Characteristics in France, Italy and the Sample as a Whole

	**Total Sample (N = 188)**				**France (n = 93)**			**Italy (n = 95)**		***P*** ** Value** ^a^
	**No. (%)**	**Mean (SD)**	**Median (Min-Max)**	**No. (%)**	**Mean (SD)**	**Median (Min-Max)**	**No. (%)**	**Mean (SD)**	**Median (Min-Max)**	
Gender										> .001
Male	64 (34.0)	-	-	31 (33.3)	-	-	33 (34.7)	-	-	
Female	128 (66.0)	-	-	62 (66.7)	-	-	62 (65.3)	-	-	
Educational level										> .001
Νon formal qualification	-	-	-	-	-	-	-	-	-	
Primary school education (up to age 12)	-	-	-	-	-	-	-	-	-	
Secondary school education (up to age 15-16)	-	-	-	-	-	-	-	-	-	
Secondary school education (up to age 18)	80 (42.5)	-	-	40 (43.0)	-	-	40 (42.1)	-	-	
University degree	42 (22.4)	-	-	21 (22.6)	-	-	21 (22.1)	-	-	
Postgraduate degree	66 (35.1)	-	-	32 (34.4)	-	-	34 (35.8)	-	-	
Age	-	50.9 (7.7)	(26-67)	-	51 (7.8)	54 (26-67)	-	50.8 (7.7)	51 (32-60)	> .001
Position in the organization										> .001
President or other board member	35 (18.6)	-	-	17 (18.3)	-	-	18 (18.9)	-	-	
Employed by the organization	43 (22.9)	-	-	22 (23.7)	-	-	21 (22.1)	-	-	
Voting member	23 (12.2)	-	-	12 (12.9)	-	-	11 (11.6)	-	-	
Non-voting but active member	50 (26.6)	-	-	24 (25.8)	-	-	26 (27.4)	-	-	
Non-active member	37 (19.7)	-	-	18 (19.3)	-	-	19 (20.0)	-	-	
Individuals’ personal involvement in the organization										> .001
Νone	6 (3.2)	-	-	3 (3.2)	-	-	3 (3.3)	-	-	
Very low	15 (8.0)	-	-	7 (7.6)	-	-	8 (8.4)	-	-	
Low	40 (21.3)	-	-	20 (21.5)	-	-	20 (21.0)	-	-	
Moderate	42 (22.3)	-	-	21 (22.6)	-	-	21 (22.1)	-	-	
High	39 (20.7)	-	-	19 (20.4)	-	-	20 (21.0)	-	-	
Very high	46 (24.5)	-	-	23 (24.7)	-	-	23 (24.2)	-	-	
Membership duration (in years)	-	4.4 (4.2)	4 (1-21)	-	4.5 (3.9)	4 (1-17)	-	4.4 (4.4)	4 (1-21)	> .001

^a^ *P* values correspond to comparisons between the samples in France and Italy.

### Statistical Analysis

Variables were firstly tested for normality using the Kolmogorov-Smirnov criterion. Variables with skewed distribution are presented with median and interquartile range (IQR). Categorical variables are presented with absolute and relative frequencies. For the comparisons of proportions, chi-square and Fisher exact tests were used were appropriate. Due to the lack of normality, the non-parametric Mann-Whitney test was computed for the comparison of median values between 2 groups, as the distribution was not normal. Also, the non-parametric Kruskal-Wallis test was used for the comparison of median values between 3 or more groups.

Exploratory factor analysis was carried out to evaluate construct validity of the HDI in the 2 samples and to determine whether the scale tapped a unidimensional construct. Principal component analysis (PCA) was chosen as extraction method using varimax rotation. The cut-off point for factor loadings was 0.4 and for eigenvalues it was 1.0. The internal consistency of the questionnaire was assessed with Cronbach α coefficient. Reliability equal to or greater than 0.7 was considered acceptable. All *P* values reported are 2-tailed. Statistical significance was set at .05 and analyses were conducted using SPSS statistical software (version 19.0).

## Results

### Psychometric Properties

The internal consistency of the HDI was considered adequate (Cronbach α = .93 in Italy and Cronbach α = .70 in France). PCA with a varimax rotation was used to explore the construct validity of the scale. One factor was revealed with eigenvalue more than one for both countries and accounted for 67.7% and 64.4% of the total variance in Italy and France respectively. All items were entered into the factor analysis and factor loadings ranged from 0.6 to 1.0. in Italian data and from 0.5 to 0.9 in French data.

### Comparison of Italy and France

The medians of the HDI items are shown in Table 2. Significantly higher values were found for all of the HDI items in the French sample as compared to their Italian counterparts.

**Table 2:** Descriptive Statistics for the HDI Items and Comparison Between Italy and France

	**France**	**Italy**	***P*** ^a^
	**Median (IQR)**	**Median (IQR)**	
*Does your patient organization participate:*			
In reforms or key decisions in health policy?	3 (2-3)	1 (1-2)	< .001
In panels of experts or workshops held in the Ministry of Health?	3 (2.5-3)	1 (1-3)	< .001
In panels or workshops in other important organizations pertinent to health?	3 (2-3)	1 (1-2)	< .001
In hospital boards?	2 (1-3)	1 (1-3)	.040
In Ethics Committees for clinical trials?	3 (2-3)	1 (1-2)	< .001
In health technology assessment procedures for the scientific evaluation of new treatments & methods?	2 (1-2)	1 (1-2)	< .001
In health technology assessment procedures for the economic evaluation of new treatments & methods?	2 (1-2)	1 (1-1)	< .001
In the national parliament during decision-making for important health policies/legislation?	2 (2-3)	1 (1-3)	< .001
*How often do you observe a substantial change in the content of a health policy decision as a result of the involvement of your patient organization?*	3 (2-5)	3 (1-4)	.009
HDI score (sum)	22 (20-27)	12 (9-17)	< .001
HDI score (average)	2.4 (2.2-3)	1.3 (1-1.9)	< .001

Abbreviations: HDI, Health Democracy Index; IQR, interquartile range. ^a^ Mann-Whitney test.

The type of participation (Table 3) was primarily “observer” in Italy, while it was “consulted” in France. As regards the “vocal participant” category, it was found to have significantly higher frequency in Italy with respect to participation in “reforms or key decisions in health policy” and “health technology assessment procedures for the economic evaluation of new treatments & methods” and significantly higher in France with regard to participation in “panels of experts or workshops held in the Ministry of Health,” “panels or workshops in other important organizations pertinent to health” and “Ethics Committees for clinical trials.”

**Table 3:** Descriptive Statistics for the Type of Participation Concerning HDI Items for Italy and France

	**Type of Participation**	**France**	**Italy**	***P***
		**No. (%)**	**No. (%)**	
In reforms or key decisions in health policy	Observer	39 (41.9)	65 (68.4)	< .001^a^
	Consulted	45 (48.4)	5 (5.3)	
	Vocal participant	9 (9.7)	20 (21.1)	
	Voting member	0 (0.0)	5 (5.3)	
In panels of experts or workshops held in the Ministry of Health	Observer	21 (22.6)	85 (89.5)	< .001^b^
	Consulted	39 (41.9)	0 (0.0)	
	Vocal participant	33 (35.5)	10 (10.5)	
	Voting member	0 (0.0)	0 (0.0)	
In panels or workshops in other important organizations pertinent to health	Observer	30 (32.3)	80 (84.2)	< .001^b^
	Consulted	42 (45.2)	0 (0.0)	
	Vocal participant	21 (22.6)	5 (5.3)	
	Voting member	0 (0.0)	10 (10.5)	
In hospital boards	Observer	42 (45.2)	80 (84.2)	< .001^a^
	Consulted	39 (41.9)	0 (0.0)	
	Vocal participant	9 (9.7)	15 (15.8)	
	Voting member	3 (3.2)	0 (0.0)	
In Ethics Committees for clinical trials	Observer	33 (35.5)	85 (89.5)	< .001^a^
	Consulted	27 (29.0)	0 (0.0)	
	Vocal participant	27 (29.0)	10 (10.5)	
	Voting member	6 (6.5)	0 (0.0)	
In health technology assessment procedures for the scientific evaluation of new treatments & methods	Observer	60 (64.5)	80 (84.2)	< .001^a^
	Consulted	24 (25.8)	5 (5.3)	
	Vocal participant	6 (6.5)	10 (10.5)	
	Voting member	3 (3.2)	0 (0.0)	
In health technology assessment procedures for the economic evaluation of new treatments & methods	Observer	78 (83.9)	80 (84.2)	< .001^a^
	Consulted	15 (16.1)	0 (0.0)	
	Vocal participant	0 (0.0)	10 (10.5)	
	Voting member	0 (0.0)	5 (5.3)	
In the national parliament during decision-making for important health policies/legislation	Observer	63 (67.7)	80 (84.2)	< .001^a^
	Consulted	27 (29.0)	5 (5.3)	
	Vocal participant	3 (3.2)	5 (5.3)	
	Voting member	0 (0.0)	5 (5.3)	

Abbreviation: HDI, Health Democracy Index. ^a^ Fisher exact test; ^b^ Pearson chi-square test.

The impact of participation was more frequently absent to low (Table 4). Additionally, it was higher in France than in Italy regarding the items “reforms or key decisions in health policy,” “panels of experts or workshops held in the Ministry of Health,” “panels or workshops in other important organizations pertinent to health,” “hospital boards,” and “Ethics Committees for clinical trials.”

**Table 4:** Descriptive Statistics for the Impact of Participation Concerning HDI Items for Italy and France

	**Impact**	**France**	**Italy**	***P***
		**No. (%)**	**No. (%)**	
In reforms or key decisions in health policy	Absent to low	45 (48.4)	75 (78.9)	< .001^a^
	Moderate	15 (16.1)	20 (21.1)	
	High/very high	33 (35.5)	0 (0.0)	
In panels of experts or workshops held in the Ministry of Health	Absent to low	42 (45.2)	65 (68.4)	.004^a^
	Moderate	39 (41.9)	20 (21.1)	
	High/very high	12 (12.9)	10 (10.5)	
In panels or workshops in other important organizations pertinent to health	Absent to low	60 (64.5)	75 (78.9)	.011^a^
	Moderate	18 (19.4)	5 (5.3)	
	High/very high	15 (16.1)	15 (15.8)	
In hospital boards	Absent to low	57 (61.3)	75 (78.9)	.010^b^
	Moderate	33 (35.5)	20 (21.1)	
	High/very high	3 (3.2)	0 (0.0)	
In Ethics Committees for clinical trials	Absent to low	60 (64.5)	80 (84.2)	< .001^b^
	Moderate	24 (25.8)	15 (15.8)	
	High/very high	9 (9.7)	0 (0.0)	
In health technology assessment procedures for the scientific evaluation of new treatments & methods	Absent to low	84 (90.3)	80 (84.2)	.495^b^
	Moderate	6 (6.5)	10 (10.5)	
	High/very high	3 (3.2)	5 (5.3)	
In health technology assessment procedures for the economic evaluation of new treatments & methods	Absent to low	69 (74.2)	70 (73.7)	.076^b^
	Moderate	24 (25.8)	20 (21.1)	
	High/very high	0 (0.0)	5 (5.3)	
In the national parliament during decision-making for important health policies/legislation	Absent to low	69 (74.2)	70 (73.7)	.246^b^
	Moderate	21 (22.6)	25 (26.3)	
	High/very high	3 (3.2)	0 (0.0)	

Abbreviation: HDI, Health Democracy Index. ^a^ Pearson chi-square test; ^b^ Fisher exact test.

In France, a statistically significant difference was documented in the HDI score among different levels of participants’ knowledge of treatment options and of their country’s healthcare system. Specifically, as shown in Table 5, participants with very low to moderate knowledge about treatment options and about their country’s healthcare system had significantly lower HDI score. Moreover a statistical significant correlation was found between participants’ contention that there is a larger umbrella organization to represent all patients or different organizations in their country and lower HDI score. A greater HDI score was found in participants who believed that their patient organization was a member of an umbrella organization that represented them on a national policy decision-making. Furthermore, a significantly greater HDI score was observed in cases where participants reported that there is a collaboration between their association and other patient organizations on a national policy/decision-making level.

In Italy, there was a significant difference in HDI score among different levels of participants’ knowledge about treatment options, their country’s healthcare system and their country’s drug reimbursement processes (Table 5). In particular, it was found that participants with very low to moderate knowledge about the aforementioned areas of knowledge had significantly lower HDI score. Furthermore, participants who reported “moderate to very high” degree of collaboration between their organization and other patient organization of the same or other disease area displayed significantly higher HDI score. Similarly, a greater HDI score was found when participants believed there is a larger umbrella organization to represent all patients or different organizations in their country as well as when they believed that their patient organization was a member of this umbrella organization.

**Table 5:** Comparisons Between France and Italy With Regard to the HDI Score

	**France**			**Italy**		
	**HDI score**					
	**No. (%)**	**Median (IQR)**	***P***	**No. (%)**	**Median (IQR)**	***P***
How would you rate your familiarity with the disease?						
Very low to moderate	6 (7.7)	21 (21-21)	.380^a^	55 (64.7)	9 (9-17)	.764^a^
High/very high	72 (92.3)	25.5 (20-27)		30 (35.3)	13 (10.5-14.5)	
How would you rate your knowledge about treatment options?						
Very low to moderate	15 (19.2)	21 (19-22)	.041^a^	55 (64.7)	9 (9-14)	.036^a^
High/very high	63 (80.8)	26 (20-27)		30 (35.3)	13.5 (10.5-23.5)	
How would you rate your knowledge about your country healthcare system?						
Very low to moderate	18 (26.1)	20 (16-21)	.001^a^	55 (68.8)	9 (9-14)	< .001^a^
High/very high	51 (73.9)	25.5 (20-28)		25 (31.3)	23.5 (15-32)	
How would you rate your knowledge about your country drug reimbursement processes?						
Very low to moderate	15 (19.2)	22 (20-26)	.373^a^	65 (76.5)	9 (9-14)	< .001^a^
High/very high	63 (80.8)	25.5 (20-27)		20 (23.5)	15 (12-32)	
Overall how would you rate the degree of collaboration of your organization with other patient organization of the same or other disease area?						
Absent to low	27 (29.0)	25 (22-26)	.472^a^	40 (42.1)	9 (9-9)	< .001^a^
Moderate to very high	66 (71.0)	21.5 (20-27)		55 (57.9)	17 (14-32)	
Is there an umbrella Patient Organization to represent all patients or different organizations in your country?						
No	63 (67.7)	26 (21-27)	.006^a^	55 (57.9)	9 (9-14)	.004^a^
Yes	30 (32.3)	20.5 (19.5-22.5)		40 (42.1)	15 (12-32)	
Is yοur patient organizatiοn a member οf an umbrella organizatiοn that represents yοu at a national policy-/decision-making level?						
No, there is no relevant federation/umbrella organization in my country	12 (12.9)	25 (16-35)	< .001^b^	25 (26.3)	9 (9-17)	.017^b^
No, my patient organization is not a member of a federation/umbrella organization	33 (35.5)	20 (19-22)		20 (21.1)	11.5 (9-14)	
Yes	45 (48.4)	27 (21-27)		45 (47.4)	15 (12-32)	
Does your patient organization collaborate with other patient organizations on a national policy-/decision-making level?						
No	21 (22.6)	19 (17.5-20.5)	< .001^a^	30 (31.6)	9 (9-17)	.071^a^
Yes	72 (77.4)	25 (21-27)		65 (68.4)	14 (9-32)	

Abbreviation: HDI, Health Democracy Index. ^a^ Mann-Whitney test; ^b^ Kruskal-Wallis test.

## Discussion

The present study aimed to validate further an original instrument for measuring patient organization participation in health policy. Moreover, by utilizing this tool, it endeavored to gauge the degree and impact of CPOs participation in France and Italy and to shed light on potential explanations for their differences.

Regarding the former aim, the instrument displayed moderate to high internal consistency in both countries (Cronbach α = .7 in France and Cronbach α = .93 in Italy). These levels are concordant with evidence emanating from the validation of the index in Greece (Cronbach α = .85 ).^31^ Furthermore, findings from factor analysis revealed that the construct of patient organization participation tapped in the instrument is unidimensional. In this reasoning, the HDI continues to display good psychometric properties, when applied in different countries.

Concerning CPOs participation in health policy, the highest level of participation in France was observed with respect to reforms and key decisions in health policy, in panels/workshops help at the Ministry of Health, in panels/workshops in other important health-related organizations and in Ethics committees for clinical trials. On the other hand, in Italy, CPOs participation is of similar degree in all aspects of health policy decision-making. It is noteworthy that the median value in all items included in the tool is below the midpoint in both countries, indicating overall low levels of CPOs participation in health policy. Similarly, in France the impact of participation was found to be higher in reforms or key decisions in health policy and in participation in panels/workshops held at the Ministry of Health or other health-related organisations. On the other hand, in Italy, the strongest impact was observed with respect to participation in panels/workshops in other important health related organizations. It is noteworthy that in both countries, the majority of participants have evaluated as absent-to-low the impact described in germane items.

When CPOs participation was compared between the 2 countries, converging data substantiate that levels of patient participation are higher in France than in Italy. In particular, France has demonstrated higher levels of participation in both item and scale analysis; while the impact of participation was also found to be greater. In addition, when the results regarding the type of participation were taken into account, the main type of participation was found to be “consultant” in France, as compared to “observed” in Italy. The differences between the 2 countries can be interpreted in light of the legislation and the preponderance of the patient-centred paradigm in the healthcare culture of each country.

The “Patients’ Rights and Quality of Care Act” in France (Act no. 2002-303) laid the foundations for promoting patients’ rights, patient empowerment and patient participation in health, facilitating their representation on a high policy level. At the same time, it paved the way for the establishment of specific guidelines and procedures for involving patients in different facets of health policy decision-making.^44^ On the contrary, in Italy there has been no specific legislation for patient participation and empowerment; even though general legislation for safeguarding patients’ rights does exist. In addition, Italy is a decentralized country and hence health policy decision-making is carried out on a regional level. As a result of this, there is a wide variation among regions with respect to the legislation on patient participation in health policy decision-making.^45^ Although the shift from central to regional level confers a lot of advantages, mainly in terms of funding and accountability, it may also cause interregional differences in patients’ involvement. Concomitantly, it has been argued that a highly decentralized system can substantially attenuate the influence of health patient organizations by diluting their capacity and diffusing further their limited resources.^46^ Apart from legislation and the configuration of the healthcare system, France has a long history in patient empowerment, especially due to the emergence of 2 generations of AIDS associations striving to achieve the active involvement of patients in health policy.^47^ It is noteworthy that in many countries, the fight against AIDS has been an exemplar of patients’ individual and collective efforts to participate in health and healthcare.^47^

It is evident that patients’ participation in health policy decision-making is facilitated by the existence of relevant legislative provisions.^3,48^ The Council of Europe clearly stipulates that patient associations should be aided to participate “in the development of policies and programs on patient safety on all appropriate levels.”^49^ Accordingly, European countries should develop appropriate legislation and procedures in order to support patient involvement. Findings from the present study have revealed that patient participation is fostered by increased levels of knowledge about the healthcare system itself and reimbursement issues. This suggests that European countries should enhance patient involvement by implementing interventions on various levels.^50^ To raise awareness and promote patient empowerment, a number of European countries have developed relevant laws and regulations; while they have organized public campaigns. Most countries have established governmental and nongovernmental organizations and have adopted a “top-down” approach to support patient participation in health decision-making.^17,51^ For instance, countries such as Australia, Canada, and the United Kingdom have established state and regional health councils, while they have developed initiatives to upgrade the role of patients in health policy decision-making.^17,52-55^ In the United States, participatory decision-making models have been developed to reinforce patient involvement. These in turn allow individuals to interact with health authorities so as to jointly formulate legislation and interventions in health related issues.^56,57^ A similar initiative in France resulted in a bill promoting a more democratic environment in health (“démocratie sanitaire”) through patient participation.^58^ The patients’ role was upgraded to include official opportunities wherein the individuals can equally contribute to and influenced decisions, policies and healthcare strategies.^58^ In the United Kingdom, the commitment of the National Institute for Health and Care Excellence (NICE) to patient and public involvement in health policy decision-making is reflected on its key principles. The NICE has also developed relevant guidelines for empowering different groups of patients as well as for supporting organizations to participate in decisions and programs pertinent to health.^59^

On the pan-European level, the “European Charter of Patients’ Rights” clearly states that patients have the right to participate in health policy-making.^60^ Likewise, according to the “Ljubljana Charter on Reforming Health Care in Europe”^61^ patients have the right to participate in shared decision-making on an equal basis and contribute to all relevant procedures that affect population health. Lastly, the Council of Europe suggests that patient empowerment should be an area of high priority.^62^

## Strengths and Limitations of the Study

The main strength of the present study pertains to the good psychometric properties of the HDI and the provision of evidence on CPOs participation in Italy and France. The good psychometric properties of the HDI suggest that the tool can be used effectively to detect different degrees of patient participation and its impact. Item drafting and selection occurred through a thorough and rigorous process. Concomitantly, the assessment of the CPOs participation in health policy decision-making processes in Italy and France provides evidence for the CPOs’ contribution to health policy on a national level, highlighting in this way the needs and deficiencies that should be addressed in order to increase their capacity to impact health programs and policies.

Apart from its strengths, the study has also certain limitations. Even though the development and psychometric testing of the HDI was based on a rigorous process, its concurrent validity could not have been examined, as there is no gold standard for assessing patient participation on a meso/macro level. Moreover, due to the fact that there is no sampling frame for CPOs in EU, different strategies for identifying CPOS and recruiting participants were employed in order to minimize sampling bias. Additionally, CPOs that did not respond to the emails, were contacted multiple times over the phone in order to ensure participation. In spite of the versatile and consistent efforts to include as many CPOs as possible, the response rate is low, with the most visible and active CPOs being included into the sample. As a corollary of this, one cannot rule out the possibility of selection bias, with those CPOs refusing to take part in the study, not responding, to have lower levels of participation in health policy as compared to the CPOs that agreed to take part. Nonetheless, as response rates are similar in both countries, it is unlikely that selection bias may explain the differences observed between the 2 countries. Furthermore, as there was no sampling frame for the study, the sample could not have been random nor representative of the population. As a result of this and in conjunction with the low response rates, findings cannot be generalized to all members of CPOs in both countries. It also merits noting that information about the capacity of CPOs was not gleaned (eg, number of members, years since establishment), as the questionnaire was already time-consuming for responders and the particular questions may have been confusing. For example upon designing the study, the research team could not easily decide on which is more important, the number of a CPO’s members is more influential or the number of its active members. Similarly, “years since the CPO’s establishment” may have not been a useful proxy of its level of activity. Nonetheless, a future study should include this type of questions in its design to ensure taking into consideration these variables as well.

The determinants for CPOs participation was not explored in depth; however, a follow-up report focusing on the individual and collective correlates of CPOs participation in both countries will be prepared.

## Suggestions for Further Research

More studies are needed in order to understand the factors that facilitate and hinder high participation of CPOs in health policy, in different countries with different legal frameworks and healthcare systems.

It is anticipated that the HDI will allow us to compare and contrast the degree of patient organization participation in decision-making in various countries and to set policy priorities in place. Moreover, the HDI can be used to evaluate the effectiveness of different programs aiming to improve patient participation in health decision-making on a meso/macro level. At the same time, it may contribute to advocacy actions by shaping recommendations for enhancing patient empowerment and optimizing patient involvement in decision-making processes.

Findings from ensuing national and international studies may lead to the development of a roadmap with the necessary steps needed so that patient organizations can establish a central position in decision-making processes in Europe.

## Acknowledgements

Research funded by Novartis Pharma, Basel, under the AGORA initiative (a European Think Tank which aims to optimize access for patients to innovative treatments). Final publication fully owned by the authors.

## Ethical issues

The study was approved by the Research and Ethics Committee of the University of Peloponnese, Corinth, Greece in accordance with the ethical standards delineated in the 1964 Declaration of Helsinki. Furthermore, the Institutional Review Board of the participating patients associations reviewed and approved the study. Informed consent for participation was obtained from all participants.

## Competing interests

Authors declare that they have no competing interests.

## Authors’ contributions

KS: conception of the study; design of the study; data interpretation; drafting the manuscript; EA: design of the study; data acquisition; data interpretation; drafting the manuscript; LEP: design of the study; data interpretation; drafting the manuscript; CT: design of the study; data analysis; data interpretation; drafting the manuscript; DV: data acquisition; data interpretation; critically revising the manuscript; OCB: data acquisition; data interpretation; critically revising the manuscript; DD: design of the study; critically revising the manuscript; SH: design of the study; critically revising the manuscript; FS: design of the study; critically revising the manuscript.

## Authors’ affiliations

^1^Faculty of Social and Political Sciences, University of Peloponnese, Corinth, Greece. ^2^Community Mental Health Centre, University Mental Health Research Institute, Athens, Greece. ^3^Department of Surgery, University of Rome Tor Vergata, Rome, Italy. ^4^Europa Donna France, Paris, France. ^5^Bulgarian National Patients’ Organization, Sofia, Bulgaria. ^6^FSNB Health & Care, Paris, France.

## Key messages

**Implications for policy makers**

- The first step to ensure patients’ participation in shaping health policies is the development and enforcement of relevant legislation.
- The main barriers preventing patients to participate in health and healthcare decisions are: limited knowledge about cancer, the health system and health policies as well as lack of lobbying and advocacy skills. Targeted interventions on these topics would be beneficial in increasing patient participation in every aspect of decision-making.
- Policy-makers should invest in regulations that focus on equal and meaningful participation of patient groups in health policy decision-making. To this end, they should develop online informational and monitoring systems.
- The Health Democracy Index (HDI) is a brief and robust tool which can be used to assess the degree and impact of patients’ organization participation in health policy decision-making.

**Implications for public**

Patient organization participation allows patients to be an integral part of the health policy decision-making course that influences their health. In recent years, patient organizations have the role of representing groups of patients at various levels (eg, local, national, international). The main objective of patient organizations is not only to participate but also to influence health policy decisions. Evidence from the present study suggests that even though there are cancer patient organizations (CPOs) in Italy and France, they fail to participate successfully in health policy decision-making processes and influence them effectively. Consistent efforts should be made on the part of citizens, patients and their representatives on acquiring a central position in health policy decision-making.

## References

[1] Longtin Y, Sax H, Leape LL, Sheridan S, Donaldson L, Pittet D (2010). Patient Participation: Current Knowledge and Applicability to Patient Safety. Mayo Clin Proc.

[2] Cahill J (1998). Patient participation - a review of the literature. J Clin Nurs.

[3] Tattersall RL (2002). The expert patient: a new approach to chronic disease management for the twenty-first century. Clin Med.

[4] Dicker A, Armstrong D (1995). Patients’ views of priority setting in health care: an interview survey in one practice. BMJ.

[5] Florin D (2004). Public involvement in health care. BMJ.

[6] Bowie C, Richardson A, Sykes W (1995). Consulting the public about health service priorities. BMJ.

[7] Thompson AG (2007). The meaning of patient involvement and participation in health care consultations: A taxonomy. Soc Sci Med.

[8] Coulter A (2002). Whatever happened to shared decision-making?. Health Expect.

[9] Coulter A (2002). Involving patients: representation or representativeness?. Health Expect.

[10] Coulter A, Parsons SJ. Where Are The Patients In Decision-Making About Their Own Care? Denmark: World Health Organization; 2008.

[11] Souliotis K. Looking for democracy in health amidst the fiscal crisis: patient participation in health policy decision making. In: Souliotis K, ed. Democracy, Citizens and Health Policy: Participation in Decision Making, Lobbying and Patient Associations (in Greek). 1st ed. Athens Greece: Papazisis; 2016:23-51.

[12] Souliotis K. The concept of Health Democracy: Documentation of a New Approach in Health Policy Decision Making. The Health Democracy Index. Paper presented at: 41 PanHellenic Medical Conference; 2015.

[13] Van de Bovenkamp HM, Trappenburg MJ (2011). Government Influence on Patient Organizations. Health Care Analysis.

[14] Ottawa Charter for Health Promotion. Ottawa, Canada: WHO; 1986.

[15] Hunter DJ. Getting knowledge on ‘wicked problems’ in health promotion into action. In Clavier C, De Leeuw E, eds. Health Promotion and the Policy Process. Oxford: Oxford University Press;2013:131-153.

[16] Hibbard JH, Greene J (2013). What the evidence shows about patient activation: better health outcomes and care experiences; fewer data on costs. Health Aff.

[17] Conklin Α, Morris Ζ, Note Ε. Involving the Public in Healthcare Policy. An Update of the Research Evidence and Proposed Evaluation Framework. A Technical Report. Santa Monica; 2010.

[18] Lenaghan J (1999). Involving the public in rationing decisions The experience of citizens juries. Health Policy.

[19] Mitton C, Smith N, Peacock S, Evoy B, Abelson J (2009). Public participation in health care priority setting: A scoping review. Health Policy.

[20] Höglund AT, Winblad U, Arnetz B, Arnetz JE (2010). Patient participation during hospitalization for myocardial infarction: perceptions among patients and personnel. Scand J Caring Sci.

[21] Weingart SN, Zhu J, Chiappetta L (2011). Hospitalized patients’ participation and its impact on quality of care and patient safety. Int J Qual Health Care.

[22] Heisler M, Bouknight RR, Hayward RA, Smith DM, Kerr EA (2002). The relative importance of physician communication, participatory decision making, and patient understanding in diabetes self-management. J Gen Intern Med.

[23] McCorkle R, Ercolano E, Lazenby M (2011). Self-management: Enabling and empowering patients living with cancer as a chronic illness. CA Cancer J Clin.

[24] Holman H. Patients as partners in managing chronic disease. BMJ 2000;320(7234):526-527. 10.1136/bmj.320.7234.526 PMC1117581 10688539

[25] Nutbeam D (2000). Health literacy as a public health goal: a challenge for contemporary health education and communication strategies into the 21st century. Health Promot Int.

[26] Hibbard J, Mahoney E, Stock R, Tusler M (2007). Do Increases in Patient Activation Result in Improved Self-Management Behaviors?. Health Serv Res.

[27] Sullivan M (2003). The new subjective medicine: taking the patient’s point of view on health care and health. Soc Sci Med.

[28] Bieber C, Müller K, Blumenstiel K (2006). Long-term effects of a shared decision-making intervention on physician–patient interaction and outcome in fibromyalgia. Patient Educ Couns.

[29] Elwyn G, Edwards A, Mowle S (2001). Measuring the involvement of patients in shared decision-making: a systematic review of instruments. Patient Educ Couns.

[30] van de Bovenkamp HM, Trappenburg MJ, Grit KJ (2010). Patient participation in collective healthcare decision making: the Dutch model. Health Expect.

[31] Raingruber B (2011). The Effectiveness of psychosocial interventions with cancer patients: an integrative review of the literature (2006–2011). ISRN Nurs.

[32] van Thiel G, Stolk P. Priority Medicines for Europe and the World. A Public Health Approach to Innovation. Update on 2004 Background Paper. Geneva: WHO; 2013.

[33] INVOLVE. Briefing notes for researchers: involving the public in NHS, public health and social care research. Eastleigh: INVOLVE; 2012.

[34] Viergever R, Olifson S, Ghaffar A, Terry RF (2010). A checklist for health research priority setting: nine common themes of good practice. Health Res Policy Syst.

[35] Wood B. Patient Power? The Politics of Patients’ Associations in Britain and America. 1st ed. Buckingham: Open University Press; 2000.

[36] Harrison S, Barnes M, Mort M (1997). Praise and damnation: mental health user groups and the construction of organisational legitimacy. Public Policy Adm.

[37] Baggott R, Allsop J, Jones K. Speaking for Patients and Carers: Health Consumer Groups and the Policy Process. 1st ed. Palgrave Macmillan; 2005.

[38] Allsop J, Jones K, Baggott R (2004). Health consumer groups in the UK: a new social movement?. Sociol Health Illn.

[39] Cancer - European Commission. Eceuropaeu. 2016. http://ec.europa.eu/health/major_chronic_diseases/diseases/cancer/index_en.htm. Accessed October 26, 2016.

[40] Jemal A, Center M, DeSantis C, Ward E (2010). Global Patterns of Cancer Incidence and Mortality Rates and Trends. Cancer Epidemiol Biomarkers Prev.

[41] Hendren S, Chin N, Fisher S (2011). Patients’ barriers to receipt of cancer care, and factors associated with needing more assistance from a patient navigator. J Natl Med Assoc.

[42] Jemal A, Ward E, Wu X, Martin H, McLaughlin C, Thun M (2005). Geographic patterns of prostate cancer mortality and variations in access to medical care in the United States. Cancer Epidemiol Biomarkers Prev.

[43] Souliotis K. The involvement of Patient Groups in health policy decision making: Pilot Study in Greece. Paper presented at: II Therapeutic Coalition Conference; 2015.

[44] Thouvenin D (2011). French Medical Malpractice Compensation since the Act of March 4, 2002: Liability Rules Combined with Indemnification Rules and Correlated with Several Kinds of Proceedings. Drexel Law Review.

[45] Ferré FB, Valerio L, Longhi S, et al. Italy: Health System Review. Copenhagen, Denmark: World Health Organization; 2014.

[46] Baggott R, Forster R (2008). Health consumer and patients’ organizations in Europe: towards a comparative analysis. Health Expect.

[47] Barbot J (2006). How to build an “active” patient? The work of AIDS associations in France. Soc Sci Med.

[48] WHO. Exploring Patient Participation in Reducing Health-Care-Related Safety Risks. Copenhagen, Denmark: WHO Regional Office for Europe; 2013.

[49] Council of Europe. Recommendation on Patient Safety, Including the Prevention and Control of Healthcare Associated Infections. Brussels, Belgium: Council of the European Union; 2009.

[50] WHO. Cross-Border Health Care in the European Union Mapping and Analysing Practices and Policies. Copenhagen, Denmark: World Health Organization on behalf of the European Observatory on Health Systems and Policies; 2011.

[51] O’Connor A, Rostom A, Fiset V (2003). Decision aids for patients facing health treatment or screening decisions: systematic review. Cochrane Database Syst Rev.

[52] Eliasoph H, Monaghan B, Beaudoin R (2007). “We are all in this together”: integrated health service plans in Ontario. HCQ.

[53] Wiseman V, Mooney G, Berry G, Tang K (2003). Involving the general public in priority setting: experiences from Australia. Soc Sci Med.

[54] Oliver S, Clarke-Jones L, Rees R (2004). Involving consumers in research and development agenda setting for the NHS: developing an evidence-based approach. Health Technol Assess.

[55] Legare F, Stacey D, Forest PG (2007). Shared decision-making in Canada: update, challenges and where next!. Z Arztl Fortbild Qualitatssich.

[56] Goold SD, Biddle AK, Klipp G, Hall CN, Danis M (2005). Choosing health plans all together: a deliberative exercise for allocating limited health care resources. J Health Polit Policy Law.

[57] Sheridan SL, Harris RP, Woolf SH (2004). Shared decision making about screening and chemoprevention. Am J Prev Med.

[58] Vega A (2014). Healthcare democracy and society’s challenges with regard to medicines (French). Soins.

[59] National Institute for Health and Clinical Excellence. Patient and Public Involvement Policy. United Kingdom: NICE; 2016.

[60] European Union. European Charter of Patients’ Rights. European Commission; 2002.

[61] World Health Organization. The Ljubljana Charter on Reforming Health Care in Europe. Copenhagen: WHO; 1996.

[62] Council of Europe. The Development of Structures for Citizen and Patient Participation in the Decision-Making Process Affecting Health Care. Strasbourg; 2000.

